# 肺癌并发异位ACTH综合征的研究进展

**DOI:** 10.3779/j.issn.1009-3419.2010.01.14

**Published:** 2010-01-20

**Authors:** 良 杨

**Affiliations:** 1 411400 湘乡，湘乡市人民医院胸外科 Department of Thoracic Surgery, People's Hospital of Xiangxiang City, Xiangxiang 411400, China; 2 310014 杭州，浙江省人民医院心胸外科 Department of Thoracic and Caridovascular Surgery, Zhejiang Provincial People's Hospital, Hangzhou 310014, China

## 1 背景资料

肺癌是当前世界各地最常见的恶性肿瘤之一，是一种严重威胁人类健康和生命的疾病。半个世纪以来，世界各国肺癌的发病率和病死率都有明显增高的趋势。近年来，我国肺癌发病率和死亡率也呈明显的上升趋势。目前肺癌总的5年生存率仍在15%以下，已成为各种癌症死亡的首要原因^[1, 2]^。世界卫生组织根据肿瘤的生物学行为和治疗、预后等因素将肺癌分为非小细胞肺癌(nonsmall cell lung cancer, NSCLC)和小细胞肺癌(small cell lung cancer, SCLC)，其中约80%-85%的肺癌为NSCLC，其病理类型主要分为鳞癌、腺癌和大细胞癌等。SCLC约占所有肺癌的15%-20%。肺癌引起的副癌综合征系肺癌的非转移性肺外表现，多为肿瘤细胞产生的某些特殊激素、抗原、酶或代谢产物所引起的临床表现，与肺癌所致的直接侵蚀、转移、阻塞无关，常累及多系统多脏器^[3]^。包括副癌神经综合征、肺原发性骨关节病、异位激素综合征等。其中异位ACTH综合征(ectopic ACTH syndrome, EAS)为肺癌患者可能并发的一类重要的副癌综合征。

异位ACTH综合征是由于垂体以外的肿瘤组织分泌过量有生物活性的促肾上腺皮质激素(adrenocorticotrophic hormone, ACTH)，刺激肾上腺皮质增生，产生过量的皮质醇而引起的临床综合征^[4]^。国外文献^[5]^报告异位ACTH综合征约占库欣综合征患者总数的10%-15%。以男性居多，男女发病比为3:1^[6]^。其最常见的病因为肺部或者支气管肿瘤，约占50%，其次为胸腺及胰腺肿瘤，各约占10%，另外有甲状腺髓样癌、纵隔肿瘤、胃肠道、生殖道等部位的肿瘤所致的EAS^[7]^。一组100例异位ACTH报道^[8]^，主要有肺癌(52%)、胰腺癌或类癌(11%)、胸腺瘤(11%)、支气管腺瘤(5%)、嗜铬细胞瘤(3%)、甲状腺癌(2%)、肝癌(2%)、前列腺癌(2%)、卵巢癌(2%)、未分化纵隔癌(2%)，乳房癌、腮腺癌、食管癌、副交感神经节瘤、交感神经节瘤各1例，另有3例原发部位不明。

1928年，美国Brown^[9]^首先报道了1例由SCLC引起的库欣综合征，从而揭开了异位ACTH激素分泌研究的序幕。Shepherd等^[10]^报告500例SCLC病人中23例(4.5%)有库欣综合征表现，其中13例在肺癌诊断时即有，10例于化疗后再次复发时出现库欣综合征。肺癌，尤其是SCLC，并发异位ACTH综合征患者的临床表现、相关机制、诊断及生化特性等有其相应特点，与肺癌患者的早期诊断、治疗和预后密切相关，因此提高对这方面的认识具有深远的临床意义。本文就肺癌并发异位ACTH综合征的相关研究进展作一综述。

## 2 临床表现

异位ACTH综合征的临床表现形式可以分为显性和隐性两种^[11]^。显性者最常见肿瘤为SCLC^[12]^，常有肾上腺皮质增生，好发于40岁以上男性，由于肿瘤恶性程度高，病情发展较快，肿瘤体积大，原发肿瘤的症状较重，容易被各种影像检查所发现。这类肿瘤的自然病程短，没有足够的时间呈现出向心性肥胖、满月脸、多血质、宽大紫纹等库欣综合征的各种典型表现，而主要表现为明显的色素沉着、高血压、水肿、严重低血钾伴肌无力，还可有烦渴、多饮、多尿、体重减轻等糖尿病症状，血浆ACTH、皮质醇显著增高。许多SCLC自然病程只有数月，临床上由于原发疾病的诊断不难，往往忽略高皮质醇血症的存在。有报道^[13]^认为肿瘤分泌的异位ACTH所造成的高皮质醇血症引起的危害远比肿瘤本身严重。由于高水平皮质醇的盐皮质激素样作用，异位ACTH综合征低血钾程度较库欣病伴低血钾患者更重^[14]^，严重低血钾可致心律失常甚至危及生命。低钾血症少见于70%的病人，并与皮质醇增多的程度相关^[15-17]^。另有报道^[18]^称低血钾碱中毒在EAS中的发生率为85%。所以对于肺癌患者应该注意异位ACTH综合征的存在。

隐性ACTH分泌瘤的恶性程度低，生长速度慢，肿瘤体积小，原发肿瘤的症状不明显，不易发现，有些原发肿瘤在库欣综合征的体征出现数年后才发现。这类肿瘤虽然恶性程度较低，但病程较长，容易发生肿瘤转移，高皮质醇血症又可引起许多并发症，预后亦不良。源自低度恶性和良性肿瘤的患者肿瘤体积较小，病程较长，病情较轻，因此临床上可表现为较典型的库欣综合征，如满月脸、向心性肥胖、紫纹、痤疮、进行性高血压、脆性糖尿病、肌无力、进行性肌营养不良、水肿及精神失常等^[18]^。

肺癌的异位ACTH综合征也可发生在呼吸道症状出现之前，时间长短不一。当肿瘤手术切除或放化疗后肿瘤缩小时，症状消失或明显缓解，肿瘤复发时，症状又可重新出现或加重。肺癌病人血清中有大量的皮质醇时容易加速转移，提示预后不良。有文献^[19]^报道，长期未能缓解的高皮质醇血症的患者的死亡率为普通人群的4倍-5倍。

## 3 相关机制

### 3.1 肺癌致异位ACTH分泌机制

Imura等^[20]^提出异位ACTH分泌肿瘤细胞主要分为3种组织病理类型：SCLC、类癌及嗜铬细胞瘤。其中SCLC引起异位ACTH分泌发病率最高，约占异位ACTH综合征病人总数的50%以上。这些肿瘤共同起源于胚胎的神经嵴，属于神经内分泌组织，具有胺前体摄取和脱羧的功能(amine precursor uptake and decarboxylation, APUD)系统。在无异位ACTH综合征临床表现的SCLC，甚至在不属于APUD系统的肺鳞状上皮癌及肺腺癌等组织提取物中也检测到ACTH肽类的表达或分泌^[21]^。近年来研究^[22-24]^发现部分NSCLC亦具有神经内分泌(neuroendocrine, NE)分化特征，结果显示，NSCLC伴NE分化的占21.24%-41.67%。张伟等^[22]^与余少平等^[23]^认为NE分化与肺癌的分化程度有密切关系，随肿瘤分化程度降低而增高，且差异具有统计学意义(*P* < 0.05)；朱维娜等^[24]^发现腺癌伴NE分化与生存时间呈负相关，与淋巴结转移和分化程度无相关性。

ACTH是由阿片-促黑素细胞-皮质素原(proopiomelanocortin, POMC)水解生成的。人类*POMC*基因为单拷贝基因，位于第2染色体(2p23)，由7 665个碱基对组成，包含3个外显子和2个内含子。在伴随有库欣综合征的异位肿瘤中，POMC mRNA表达量比垂体低，估计与分泌颗粒大量迅速分泌出细胞外有关，其mRNA长度有1 450 bp和1 200 bp左右两种。POMC基因中存在上、中、下游3个启动子：P1、P2、P3。糖皮质激素负反馈调节的蛋白结合位点在POMC基因P2启动子位置，而P1启动子调控合成的多肽产物不受正常水平糖皮质激素抑制，是导致异位ACTH综合征的可能机制之一。P3启动子调控转录的mRNA因缺乏信号肽不能翻译或部分翻译后，终产物难以分泌出细胞外，所以正常外周组织的*POMC*基因表达基本不影响正常内分泌功能。国外对*POMC*基因上存在3个不同启动子的现象称之为“promotor switch”(启动子开关)^[25]^。关于是否确实存在一个控制启动子开关的因素，还是因为不同的基因表达起源于不同的细胞亚群目前没有阐明，这一现象的临床意义有待深入研究。

美国Odell教授^[26]^提出的激素合成脱抑制学说认为，“异位激素分泌”实质上是在许多正常组织中存在的，但量极微，不会导致临床表现，而在致癌因素诱导下，由于基因表达的脱抑制而某种激素合成增加，即所谓“放大体系”，这样就有可能引起相应的临床表现。分子水平对肿瘤起源的研究主要集中在染色体缺失、基因突变及癌基因过量表达等方面。以SCLC为例，陆续发现该类细胞第3染色体短臂21区(3p21)缺失、*p53*基因突变^[27]^、*myc*、*jun*、*fos*等癌基因过量表达，这一点与垂体ACTH瘤类似。

### 3.2 皮质醇致低钾机制

1978年Arriza等研究发现皮质醇、醛固酮二者与盐皮质激素受体的亲和力相同。但在生理情况下氢化可的松仅具有弱的盐皮质激素样作用。Quinkler等^[28]^研究发现11β-羟类固醇脱氢酶主要存在于盐皮质激素反应组织，紧邻盐皮质激素受体。生理情况下，糖皮质激素在与盐皮质激素受体结合前，大多被邻近受体的11β-羟类固醇脱氢酶所代谢，很少通过与盐皮质激素受体结合而发挥盐皮质激素样作用。库欣综合征发生低钾血症可能与11β-羟类固醇脱氢酶“缺乏”有关^[29, 30]^，当体内皮质醇水平明显增高时，超过11β-羟类固醇脱氢酶对其的代谢能力，未被代谢部分则与盐皮质激素受结合，从而促进肾远曲小管Na^+^和K^+^交换，引起水钠潴留和钾丢失。11β-羟类固醇脱氢酶的这种缺乏并非真正的缺乏，而是相对于升高的皮质醇水平来说是不足的。

Arteaga等^[31]^通过对两种ACTH升高的病人(异源性ACTH综合征和17-羟化酶缺乏症)的研究发现，11-去氧皮质酮(deoxycorticosterone, DOC)、皮质酮升高造成了患者的低钾血症碱中毒，认为DOC和皮质酮分泌增加与电解质异常的关系更密切。而White等^[32]^通过对异源性ACTH综合征病人的研究认为，在ACTH的强烈刺激下，类固醇合成途径中各种酶的反应程度不同，可能造成某种(或某些)中间产物的积聚(如11β-羟化酶)，从而出现DOC积聚，但当皮质醇大量分泌且外周代谢不完全时，它具有100%的醛固酮样作用，故DOC在低钾血症的发生中只起一种附加作用。

总之，库欣综合征发生低钾血症与皮质醇的分泌量有关，发生机制可能是11β-羟类固醇脱氢酶相对不足。

## 4 诊断方法

异位ACTH综合征诊断标准：①库欣综合征的临床表现；②血皮质醇水平较正常显著增高且生理波动消失；③血ACTH水平明显高于正常；④影像学发现肾上腺外占位且垂体CT或MRI阴性，或者手术切除占位病变组织学检查发现该病变分泌ACTH。在肺癌患者同时伴有较严重的低血钾、碱中毒、高血压、糖耐量异常、水肿、肌无力、肌萎缩等症状时，应考虑异位ACTH综合征，并进行相关检查。

### 4.1 血和尿的皮质醇测定

诊断皮质醇增多症最直接和可靠的指标是测定24 h尿皮质醇含量(24 hUFC)，其可避免血皮质醇的瞬时变化，也可避免受血中皮质类固醇结合球蛋白(CBG)浓度的影响，对库欣综合征的诊断有较大的价值，诊断符合率约为98%，但一定要准确留取24 h尿量，并且避免服用影响尿皮质醇测定的药物。对于血浆皮质醇水平测定，因皮质醇呈脉冲式分泌，且血浆皮质醇水平的测定极易受情绪、应激状态、静脉穿刺是否顺利等因素影响，故单次测定血浆皮质醇水平对本病诊断的价值不大。而测定皮质醇昼夜分泌节律的消失比清晨单次测定血浆皮质醇水平有意义。有学者推荐作连续2 d-3 d测定后取平均值^[33]^。

### 4.2 血ACTH测定

血浆ACTH水平测定对鉴别ACTH依赖性和非依赖性有肯定的诊断意义^[34]^，但对鉴别是来源于垂体性还是异位的ACTH分泌增多却仅能作为参考。判断血ACTH值应结合血皮质醇的测定值。因为血皮质醇可抑制血ACTH，故当血皮质醇很高时，血ACTH处在正常范围上限即可认为有所升高。

### 4.3 胸部影像学检查

因异位ACTH综合征临床表现可以首发，且异位ACTH分泌瘤的高发区是胸部，拍摄胸片、胸部CT已成为常规检查项目。如果临床上高度怀疑异位来源，可以行胸部5 mm断层CT扫描，如果仍是阴性可行增强扫描或MRI。异位ACTH肿瘤常常表达生长抑素的受体，因此约有80%的异位库欣综合征的患者可以通过同位素标记的奥曲肽扫描发现并定位肿瘤^[35]^。

### 4.4 病理组织学

随着免疫组化技术的应用，对细胞某些特殊表征的鉴定越来越方便。ACTH、嗜铬蛋白A(CgA)、神经特异性烯醇化酶(NSE)等免疫组化已应用于临床异位ACTH综合征病理诊断。当手术切除的标本经免疫组化检查证明ACTH阳性，并且术后的血浆ACTH浓度降至正常时，对异位ACTH源的搜索才能停止，否则应考虑还有其它部位的肿瘤^[36]^。

## 5 治疗

肺癌并发异位ACTH综合征最好的治疗方法是切除原发肿瘤，如果肿瘤已有转移，也应将原发肿瘤及转移灶尽可能切除干净，手术以后再加局部放疗，必要时用药物治疗。手术后局部放疗加药物治疗使病人的存活时间明显延长。早期诊断，手术切除的成功机会极大。但某些无法切除者，可选用化疗和/或放疗。

### 5.1 围术期处理

此类患者全身状况差，病情复杂，常涉及多器官功能，麻醉管理要重视术前补钾、降糖，改善全身状况，麻醉用药少量多次，术中监测血气和ACTH、皮质醇，术中、术后应用氢化考的松预防皮质醇危象，协助患者安全度过围手术期。高皮质醇血症淋巴细胞减少，抗体形成抑制，易发生各种感染，尤其是肺感染，全麻插管后也易诱发肺部感染。因此术后雾化吸入，鼓励患者做深呼吸、咳嗽、咳痰，协助其翻身叩背，可促进肺功能恢复和预防肺部感染^[37]^。

### 5.2 药物治疗

酮康唑(ketoconazole)已成功用于治疗SCLC引起的异位ACTH综合征^[38, 39]^。酮康唑的作用是抑制线粒体细胞色素P450依赖酶，包括11β羟化酶和胆固醇碳链酶，从而阻断肾上腺类固醇侧链，抑制皮质醇的产生，使病人在1周-2周内发生急性肾上腺皮质功能减退，开始可用酮康唑800 mg/d，以后减量，以维持正常皮质醇水平，避免复发。长期使用应注意监测肝功能，可用于术前准备或联合治疗。由于患者易伴发感染，在化疗前应尽早使用酮康唑等抑制过量皮质醇产生，以预防致命的感染^[40, 41]^。同时要注意化疗致瘤体细胞破裂，可能释放大量ACTH而使化疗后异位ACTH综合征症状加重。另外可试用生长抑素类似物奥曲肽，在某些情况下，可能是相对有效的长期治疗手段，但报道较少，尚难评定确切疗效^[35]^。

综上所述，这类病人在临床中常常会忽略异位ACTH综合征的诊断，因而不会去纠正因高皮质醇血症带来的一些加速病人死亡的问题，这对于严重的难于纠正的低钾血症患者尤为重要。因此，临床医生应当主动和内分泌医生合作，在临床上对于血钾持续不升者，应及时检测患者血钾、24 h尿钾排出量、血浆ACTH和皮质醇水平，提高异位ACTH综合征的诊治水平，以指导临床治疗。
